# The Visual Analogue Scale for Rating, Ranking and Paired-Comparison (VAS-RRP): A new technique for psychological measurement

**DOI:** 10.3758/s13428-018-1041-8

**Published:** 2018-04-17

**Authors:** Yao-Ting Sung, Jeng-Shin Wu

**Affiliations:** 1 Department of Educational Psychology and Counseling/Chinese Language and Technology Center, National Taiwan Normal University, Taipei, Taiwan; 2 Research Center for Psychological and Educational Testing, National Taiwan Normal University, New Taipei City, Taiwan

**Keywords:** Likert-type scale, Paired comparison, Ranking, Multi-item VAS, VAS-RRP, CTCU model

## Abstract

Traditionally, the visual analogue scale (VAS) has been proposed to overcome the limitations of ordinal measures from Likert-type scales. However, the function of VASs to overcome the limitations of response styles to Likert-type scales has not yet been addressed. Previous research using ranking and paired comparisons to compensate for the response styles of Likert-type scales has suffered from limitations, such as that the total score of ipsative measures is a constant that cannot be analyzed by means of many common statistical techniques. In this study we propose a new scale, called the Visual Analogue Scale for Rating, Ranking, and Paired-Comparison (VAS-RRP), which can be used to collect rating, ranking, and paired-comparison data simultaneously, while avoiding the limitations of each of these data collection methods. The characteristics, use, and analytic method of VAS-RRPs, as well as how they overcome the disadvantages of Likert-type scales, ranking, and VASs, are discussed. On the basis of analyses of simulated and empirical data, this study showed that VAS-RRPs improved reliability, response style bias, and parameter recovery. Finally, we have also designed a VAS-RRP Generator for researchers’ construction and administration of their own VAS-RRPs.

Likert-type scales are one of the most popular rating scales used in surveys to measure respondents’ traits. They typically have three or more response categories to choose from, and respondents select the category that reflects their state and trait best (Likert, 1932). However, Likert-type scales have some inherent disadvantages, such as response styles, the fact that they produce ordinal measurement data, and ambiguous numbers of response categories, which prevent the accurate identification of respondents’ latent traits, and also adversely affect the use of statistical analysis methods and subsequent results (Allen & Seaman, 2007). Response styles are the systematic tendencies of respondents in their choices of certain response options (Paulhus, 1981). For example, respondents are inclined to select either neutral or middle response categories (Albaum, 1997; Greenleaf, 1992) or to provide extreme responses (Greenleaf, 1992). These response styles will lead to biased answers, which prevent the respondents’ true characteristics or traits from being obtained (Paulhus, 1981, 1991).

The psychometric property of Likert-type scales is another issue. Likert-type scales are an ordinal-level measure but not an interval-level measure—that is, the response categories have a rank order, but the intervals between values cannot be presumed to be equal (Jamieson, 2004). Ordinal data are usually described using frequencies of responses in each category, and thus the appropriate inferential statistics for ordinal data are those employing nonparametric methods, but not parametric methods, which require interval data (Allen & Seaman, 2007; Bollen, 1989; Jamieson, 2004). Many researchers ignore the problems of Likert-type scales all together and avoid mentioning them, such as by treating their ordinal data as interval and summing up the subscales (Tabachnick & Fidell, 2001). However, using ordinal data with statistical procedures requiring interval-scale measurements causes problems. For example, Bollen and Barb (1981) showed that estimates of the Pearson correlation coefficient are underestimated when computed for ordinal data. Babakus, Ferguson, and Jöreskog (1987) found that using ordinal data generally led to underestimating the factor loadings and overestimating their standard errors. Specifically, the biases induced by using various amounts of ordinal data points to calculate means, covariance, correlations, and reliability coefficients were derived by Krieg (1999), and he concluded that the more points the better, with a continuous scale being the optimal choice.

Furthermore, researchers hold a wide variety of views on how to determine the appropriate number of response categories for Likert-type scales to use in measurement (Alwin, 1992; Cox, 1980; McKelvie, 1978; Preston & Colman, 2000; Viswanathan, Bergen, Dutta, & Childers, 1996). Alwin (1992) argued that scales with more response categories are more reliable and more valid. Using only a few response categories restricts respondents’ ability to precisely convey how they feel (Viswanathan et al., 1996). In contrast, McKelvie (1978) pointed out that a relatively small number of response categories (five or six) should be used for ease of coding and scoring, and such a format will not significantly reduce reliability. Besides, both Ferrando (2003) and Scherpenzeel and Saris (1997) suggested that the number of response categories used by respondents depended on many factors, such as the type of scale, and respondents’ motivational and cognitive characteristics. These studies with ambiguous or conflicting conclusions make selecting an appropriate number of response categories quite an ordeal. In fact, there may be no optimal number of response alternatives, because regardless of the amount the researcher will still encounter serious issues.

For those who do not wish to ignore the problems inherent to Likert-type scales, there are several approaches to improving their use. The first approach involves using different data collection procedures or different scale formats to measure the respondents’ traits. For example, a comparison, or ipsative, method was proposed to reduce response-style biases because in comparison methods respondents cannot endorse every item, and consequently may eliminate uniform biases such as acquiescent responding (Cheung & Chan, 2002; Cunningham, Cunningham, & Green, 1977; Greenleaf, 1992). Meanwhile, visual analogue scales (VAS) are scales developed to obtain measurements with more variability, and use a line continuum instead of the five or seven categories used by Likert-type scales to measure latent traits (Flynn, van Schaik, & van Wersch, 2004; Guyatt, Townsend, Berman, & Keller, 1987; Jaeschke, Singer, & Guyatt, 1990). Researchers claimed that allowing participants to place their responses anywhere on a continuous line not only makes VAS free from the problem of determining the number of response categories, but also produces continuous- and interval-level measurement data (e.g., Myles, Troedel, Boquest, & Reeves, 1999; Price, McGrath, Rafii, & Buckingham, 1983; Reips & Funke, 2008). The third approach involves using mathematical transformation methods to rescale ordinal data into interval data and remedy the psychometric issue of Likert-type scales. After transformation, ordinal Likert data were able to be used in the application of suitable statistical techniques for further analysis (Chimi & Russell, 2009; Cook, Heath, Thompson, & Thompson, 2001; Granberg-Rademacker, 2010; Harwell & Gatti, 2001).

Nevertheless, although the aforementioned approaches have overcome parts of the disadvantages of Likert scales, they all introduced their own problems (see the next section). The most ideal method, thus, may be to use a scale that is able to collect fine-grained data, and is also able to avoid measurement errors and additional transformation processes, and forestall the potential problems with absolute judgments. Moreover, the new scale should be equipped with a comparison function to reduce response-style biases. Based on these ideas, the first purpose of this study is to propose the Visual Analogue Scale for Rating, Ranking, and Paired-Comparison (VAS-RRP) for data collection, to ameliorate the measurement quality of ranking, paired comparison, and Likert-type scales through use of multi-item VAS (see The VAS-RRP section). The second purpose of the study is to empirically evaluate the reliability, and parameter recovery of the VAS-RRP through simulation and empirical studies.

## Literature review

### The comparison method approach to improving the Likert-type scale

Many other methods have been proposed to tackle the disadvantages of Likert-type scales. The first and most commonly used method is adopting a forced-choice method, such as ranking or paired comparison, to reduce response-style bias. The method of ranking is based on how a respondent ranks multiple items according to a certain criterion or quality. Consider the ranking of personal preferences as an example. The respondent could rank four different items {A, B, C, D} in a single list from the most to the least favorite in the following order: B, C, A, and D. Paired comparison, on the other hand, would group the items in pairs for the comparison: in this case the four items {A, B, C, D} would be grouped as {A, B},{A, C}, {A, D}, {B, C}, {B, D}, and {C, D}. The respondent is then asked to compare each pair separately in terms of personal preferences. Many studies have pointed out that using ranking or paired comparison can effectively resolve the response style problem of Likert-type scales because comparison methods do not allow the endorsement of every item, and thus eliminate uniform biases such as acquiescent responding (Baron, 1996; Cunningham et al., 1977; Greenleaf, 1992; Randall & Fernandes, 1991). Ranking and paired comparison have been adopted by numerous scales and inventories, such as the Gordon Personal Profile Inventory (Gordon, 1993), the Minnesota Importance Questionnaire (Gay, Weiss, Hendel, Dawis, & Lofquist, 1971), the O* NET Computerized Work Importance Profiler (McCloy et al., 1999a), and the Kolb Learning Style Inventory (Kolb, 2005).

Although ranking and paired comparison may reduce the response-style bias associated with Likert-type scales, they have their own problems. As the number of items increases, paired comparison becomes extremely time-consuming and laborious for participants (Rounds, Miller, & Dawis, 1978). The number of judgments increases very rapidly as the number of items increases. From a mathematical point of view, paired comparison and ranking are ipsative measures, and this creates analytical problems or problems related to interpretation (Hicks, 1970; Meade, 2004). For example, the mean, standard deviation, and correlation coefficient of an ipsative measure cannot be used for comparison or interpretation purposes because these values merely represent the ranking of the variables. Moreover, because the sum of the item scores is a constant, as each of the rows and columns of a covariance matrix sums to zero, the covariance matrix is singular, and hence does not have an inverse matrix. This means that many statistical methods (e.g., factor analysis) that use covariance matrices for analysis become inapplicable. Also, when the sum is a constant, it turns the positive correlation between some variables into a negative correlation (Clemans, 1966; Dunlap & Cornwell, 1994).

There have been many attempts to solve the problems associated with ipsative measures. Jackson and Alwin (1980) suggested a way to transform ipsative measures, based on the assumption that an ipsative measure is obtained by subtracting the mean from the original data values. However, not all ipsative measures are obtained this way; for example, ranking involves comparing items instead of subtracting the mean, and hence the method suggested by Jackson and Alwin only works for certain types of ipsative measures. Other attempted solutions include Chan and Bentler (1998), who proposed a method based on covariance structure analysis for ranking data, and Brown and Maydeu-Olivares (2011, 2013), who re-parameterized the second-order Thurstonian factor model into a first-order factor model and proposed using the Thurstonian IRT model to analyze ranking and paired comparison data. However, the statistical techniques of Brown and Maydeu-Olivares (2011, 2013) are subject to limitations in practice. For example, their algorithms cannot handle inventories that include a larger number of items (e.g., 23 blocks with 138 items) at the same time, because large quantities of items cause huge numbers of comparisons and even more estimated parameters, which surpass the handling capacity of their algorithms.

### Using visual analogue scales to improve the Likert-type scale

Another major issue with the use of Likert-type scales is the ambiguous number of response categories. One commonly used method for avoiding this disadvantage is using a VAS (Flynn et al., 2004; Guyatt et al. 1987). A VAS is typically presented as a horizontal line, anchored with two verbal descriptors at the extremes where respondents indicate their perceived status by placing a mark along the horizontal line at the most appropriate point (Wewers & Lowe, 1990). VASs are easy to understand, administer, and score, especially when the VAS is implemented with a computer (Couper, Tourangeau, Conrad, & Singer, 2006; Wewers & Lowe, 1990; Yusoff & Janor, 2014). There are several important psychometric features of a VAS. First, the line continuum of a VAS enables the rater to make more fine-grained (Chimi & Russell, 2009) responses without the constraints of direct quantitative terms (Wewers & Lowe, 1990), and thus measurement data with higher variability will be obtained, which theoretically enhances their reliability (Cook et al., 2001; Krieg, 1999). This resolves the drawback of Likert-type scales, which have coarse-grained discrete measurement data produced by only three to seven categories. Second, VAS may provide interval-level measurements that are eligible for more statistical operations. The interval-level scale can be defined as a numeric scale on which people may assign numbers to objects in such a way that numerically equal distances on the scale represent equal distances between the features/characteristics of the objects being measured. Researchers have provided evidence for the interval-level measurement of VAS (e.g., Price, McGrath, Rafii, & Buckingham, 1983). Recently, Reips and Funke (2008) designed experiments based on judgments of equal intervals in psychophysics (Stevens, 1946, 1951) and provided evidence that participants’ responses to a VAS possess the property of an interval-level scale. Third, because of the high variability of a VAS, researchers and practitioners need not bother to determine the number of response categories (Flynn et al., 2004; Funke & Reips, 2012; Guyatt et al., 1987; Jaeschke et al., 1990; Kuhlmann, Dantlgraber, & Reips, 2017).

Despite the advantages mentioned above, several features of VASs need to be investigated. For example, whether the reliability and validity of VASs outperform those of Likert-type scales remains controversial, especially when different delivering tools are involved (e.g., computer-based vs. paper-and-pencil; Couper et al., 2006; Kuhlmann et al., 2017; Wewers & Lowe, 1990). Furthermore, most VASs have been administered in the format of a single item coupled with a single question; that is, each item was composed of a target attribute (or trait, statement, description, question, etc.) to be rated, along with the line continuum. This may result in absolute judgments along the continuous scale, and thus unsatisfactory reliability (e.g., Ferrando, 2003; Munshi, 2014). Both psychologists and psychometricians (e.g., Laming, 2004; Nunnally, 1967) have proposed that humans are much better at making comparative judgments than at making absolute judgments. Since multiple attributes can be located on the line continuum of a VAS simultaneously, for both ranking and paired comparison, the feasibility and psychometrical properties of using a VAS for ranking and paired comparison are worthy of investigation, especially because doing so would effectively duplicate all of the functionalities present in Likert-type scales.

### Using transformations to address issues with Likert-type scales

To overcome the psychometric issues of Likert-type scales, several researchers (e.g., Granberg-Rademacker, 2010; Harwell & Gatti, 2001; Wu, 2007) have proposed transformation methods to scale ordinal Likert-type data before statistical estimation or hypothesis testing. These methods utilize different mathematical models and mechanisms to rescale ordinal Likert-type data to interval data. For example, Harwell and Gatti applied item response theory (IRT) to model the discrete total scores obtained by test-takers to an interval-scaled proficiency. They argued that a nonlinear transformation of the IRT method would produce data that are not only interval-scale measures but also approximately normally distributed and suitable for statistical procedures. Additionally, recently Granberg-Rademacker proposed a Markov chain Monte Carlo scaling modeling technique method that converts ordinal measurements to interval. Finally, Wu applied Snell’s method to transfer 4- and 5-point Likert-type scales to numerical scores. Snell’s method assumes an underlying continuous scale of measurement and that the underlying continuous distributions follow a logistic function. Wu argued that the transformed data better followed the assumption of normality.

However, even researchers adopting such transformation approaches have acknowledged the complexity and difficulty of their transforming operations (e.g., Harwell & Gatti, 2001; Wu, 2007); because these procedures require extensive mathematical and statistical professional knowledge, the transformations are complicated to handle for people without a background in statistics or psychometrics. Moreover, mathematical models with many additional assumptions are required when applying the transformations. Those different mechanisms underlying the mathematical models make it difficult to evaluate the accuracy of the data after the transformation (Yusoff & Janor, 2014). In addition, the improvement offered by such transformations is uncertain; for instance, many indices of factor analysis have not demonstrated much difference between Likert-type scales and transformed Likert-type scales (Wu, 2007).

## The VAS-RRP

### Components of the VAS-RRPs and their usage

**Fig. 1:**
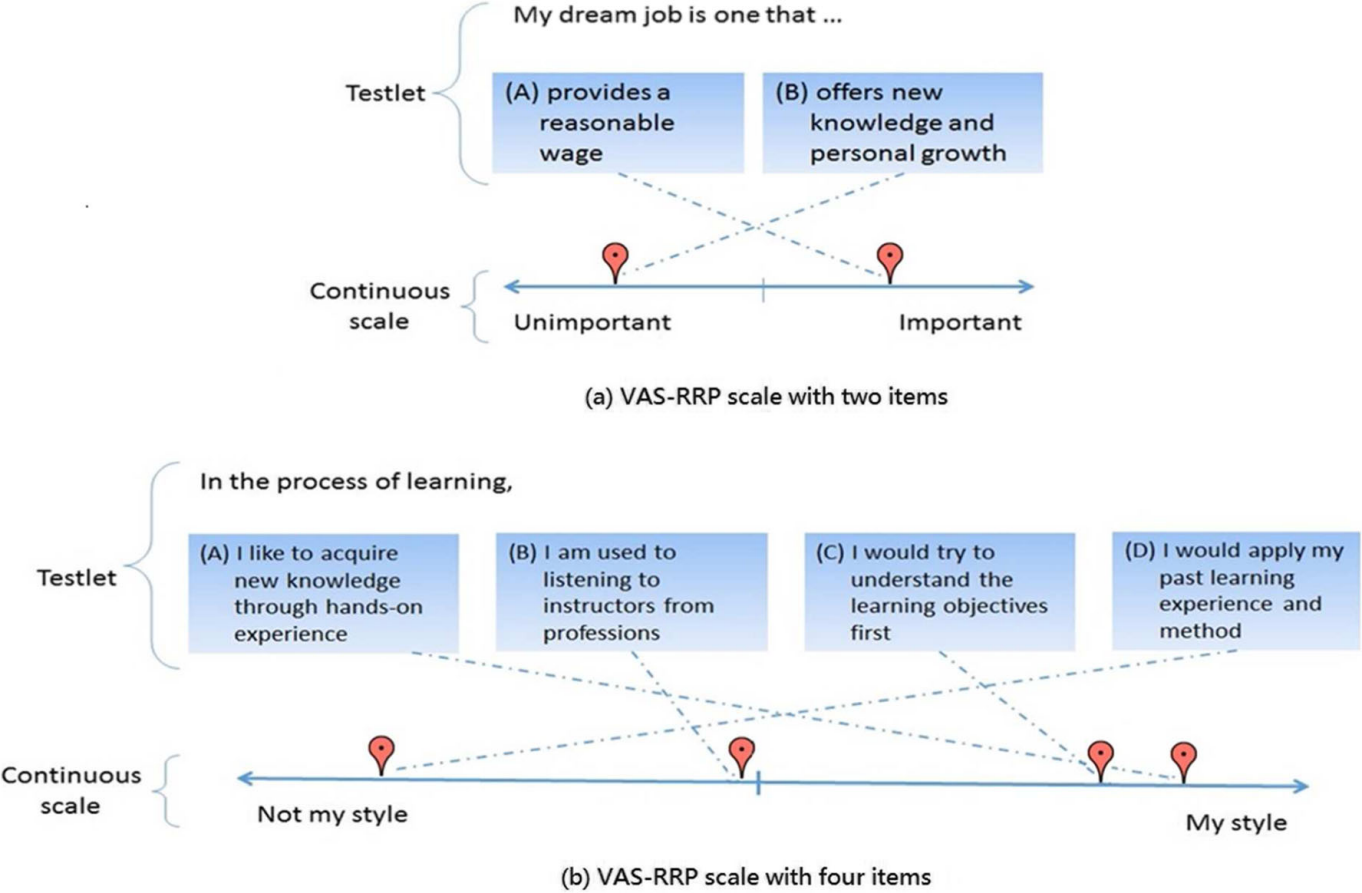
Two examples of the Visual Analogue Scale for Rating, Ranking, and Paired-Comparison (VAS-RRP) after a user has placed each item on the continuum

VAS-RRPs consist of two components (Fig. 1): The first is a testlet, composed of one or more items, which may be of one or several semantic types—such as adjectives, nouns, phrases, and sentences—for eliciting participants’ internal responses, including attitudes, opinions, interests, and so forth. The second component is a continuous rating scale, which is a line continuum with a midpoint and two directional arrows referring to two increasingly opposite levels of semantics; for example, indications made toward the right of the continuum reflect a respondent with a higher level of preference for certain objects, whereas those made toward the left reflect increasing aversion.

While using a VAS-RRP, if there is only a single item in a testlet, the respondent first checks the item and then indicates its appropriate position on the line continuum by dragging and dropping the item onto the scale, which is similar to the response to an item on a typical VAS. If there are multiple items in a testlet, respondents can repeat the procedure described above for a single item several times, until all the items in the testlet are located on the line continuum. During the process, respondents are allowed to move any item freely on the line and to do plenty of comparisons, until the relative positions of all items on the line match up to the respondent’s opinions. Meanwhile, different items in the testlet are not allowed to be marked at the same point, which assures that the VAS-RRP can be used as a comparison method. The scores of each item are calculated on the basis of the coordinates on the line continuum, which are represented by the pixels on the computer screen. Specifically, if *x*_1_ and *x*_2_ represent the two endpoint coordinates on the continuum, and the respondent indicates an item as *x*_3_, the score is calculated as $\frac{x_3-{x}_1}{x_2-{x}_1}$ for the item, which ranges from 0 to 1, indicating the level of intensity or strength of the item. Note that linear transformations can be used. For example, scores can be adjusted to fall within the range of [0, 100], or moved horizontally to an interval with 0 as the midpoint, such as [–1, 1]. Because the value for a participant’s response can be any number within the chosen range, a VAS-RRP, like a VAS, can be considered a very fine-grained scale.

Figure 1 shows two example VAS-RRP scales. Figure 1a is a testlet with two items. The respondent compares the two items on the basis of their perceived importance, and then indicates the items on the continuum. In addition, the midpoint of the continuum helps the respondent differentiate whether or not an item is considered important. In Fig. 1a, the respondent indicated that one item is important and the other is not. Figure 1b is a testlet that has four items (A, B, C, and D) representing four different styles of learning. The respondent has rated how similar each of the learning styles is to his or her own personal learning. The respondent lists the styles as A, C, B, and D, in order of decreasing similarity with his/her own ways of learning. The figure shows that the respondent considers A and C to be quite similar to his/her learning style, whereas B and D are not. Note that the respondent’s indication of B is closest to the midpoint of the continuum. The figure also shows that the respondent considers the difference between A and C to be slight, and the differences between B and C and between B and D to be larger.

### Features of the VAS-RRP

As compared to Likert-type scales, ranking, paired-comparison methods, and VASs, VAS-RRPs have distinct features, as follows:

1. Similar to the response format of the VAS, the VAS-RRP can elicit respondents’ fine-grained responses on a line continuum.
2. In the response format of a VAS-RRP with multiple items in each testlet, respondents can implement comparative judgments for the items in each testlet. Compared with the criticized “absolute judgment” function of a single-item VAS (Goffin & Olson, 2011) and Likert-type scales (Sheppard, Goffin, Lewis, & Olson, 2011), the comparative judgment function of VAS-RRPs not only provides respondents with a more authentic measurement tool for human judgments (Laming, 2004; Nunnally, 1967) but also realizes the ideal of collecting more diverse types of data, such as rating, ranking and paired comparison, in a single operation.
3. Although VAS-RRPs can be implemented in a context of comparisons, the total score of the summed items is not a constant, which is different from the traditional ipsative scales with the same total summed scores (i.e., a constant). Thus, many statistical procedures that cannot be administered to ipsative data can be applied to VAS-RRP-produced measurements. Furthermore, as compared with ranking or paired comparisons, which may only produce qualitatively different information among items (e.g., A > B > C) after certain transformation methods (e.g., Granberg-Rademacker, 2010; Harwell & Gatti, 2001; Wu, 2007), VAS-RRPs can not only provide this qualitative information, but also quantify the degree of difference among those items, because the position of each item on the line continuum is clearly indicated and on the same spectrum. This quantitative information will not only help researchers find out the exact differences among ranked items, but also help clearly identify the inclination of a participant’s attitude (e.g., positive or negative, like or dislike, important or unimportant), which can be shown by observing if the averaged scale score is above or below the midpoint. Such clarification is important for scales such as work values or career interest; however, it cannot be achieved through ranking or paired comparison, because those methods do not have a reference point for comparisons (McCloy et al., 1999a).
4. Other types of scales can be viewed as special cases of the VAS-RRP. For example, if the VAS-RRP has only one item in each testlet, the VAS-RRP can be used as a graphic rating scale or a VAS; this format of VAS-RRP can also be used as a Likert-type scale by assigning categories (e.g., five or seven terms for describing the intensity) to the line continuum for responses and calculating the scores. For the format of a VAS-RRP with two or more items, the VAS-RRP can function as a ranking or paired-comparison task, because the ordering positions of all those items on the line continuum reveal information about ranks, and the relative positions of each item reveal information about paired comparisons. Moreover, using a VAS-RRP for implementing paired-comparison tasks reduces the load for respondents, in contrast to the traditional paired-comparison task, in which $\left(\genfrac{}{}{0pt}{}{n}{2}\right)$ numbers of item comparisons are needed. With VAS-RRP the respondent only needs to read the items on a testlet and consider their relative positions on the line continuum, which saves time and energy.

### Analysis of VAS-RRP

Specifically, in data from VAS-RRPs with multiple items, the scores of each item will be affected by three factors: latent variables, measurement error, and the context effects of comparisons, which are the mutual influences of the items in the same testlet. Although the design of the testlets will help respondents make comparative judgments and might avoid response-style biases, it is noteworthy that when the procedure of model fitting is applied, the context effect within a testlet may reduce the accuracy of the parameter estimations (Holyk, 2008). However, we can take context effects into account in statistical analyses in order to obtain more accurate results. For example, the correlated-traits–correlated-uniqueness model (CTCU model; Marsh, 1989; Marsh & Bailey, 1991) is one of the statistical models that can be applied to take the contextual factors into account.

The CTCU model, developed for confirmatory factor analysis (CFA), has been primarily used for multitrait–multimethod (MTMM) data processing (Marsh & Bailey, 1991). It sets correlated trait factors, whereby method effects are inferred from correlations of the error terms (Tomás, Oliver, & Hontangas, 2002). As compared with the trait-only model (the CT model), which posits trait factors but no method effects, the CTCU model infers the method effects from the correlated uniqueness among the measured variables on the basis of the same methods (Marsh & Grayson, 1995). Adopting the idea from CTCU, in the present study we inferred the item score correlations and context effects that resulted from interitem comparisons in the same testlet from the correlations of measurement errors. Another reason for applying the CTCU model is that incorrect solutions are less likely to occur during the analysis process of model fitting (Marsh, 1989; Tomás et al., 2002), such as when the variance is < 0 or the correlation is > 1 or < – 1. The software LISREL or Mplus can be utilized directly to estimate parameters or evaluate the goodness of fit of the model.

**Fig. 2:**
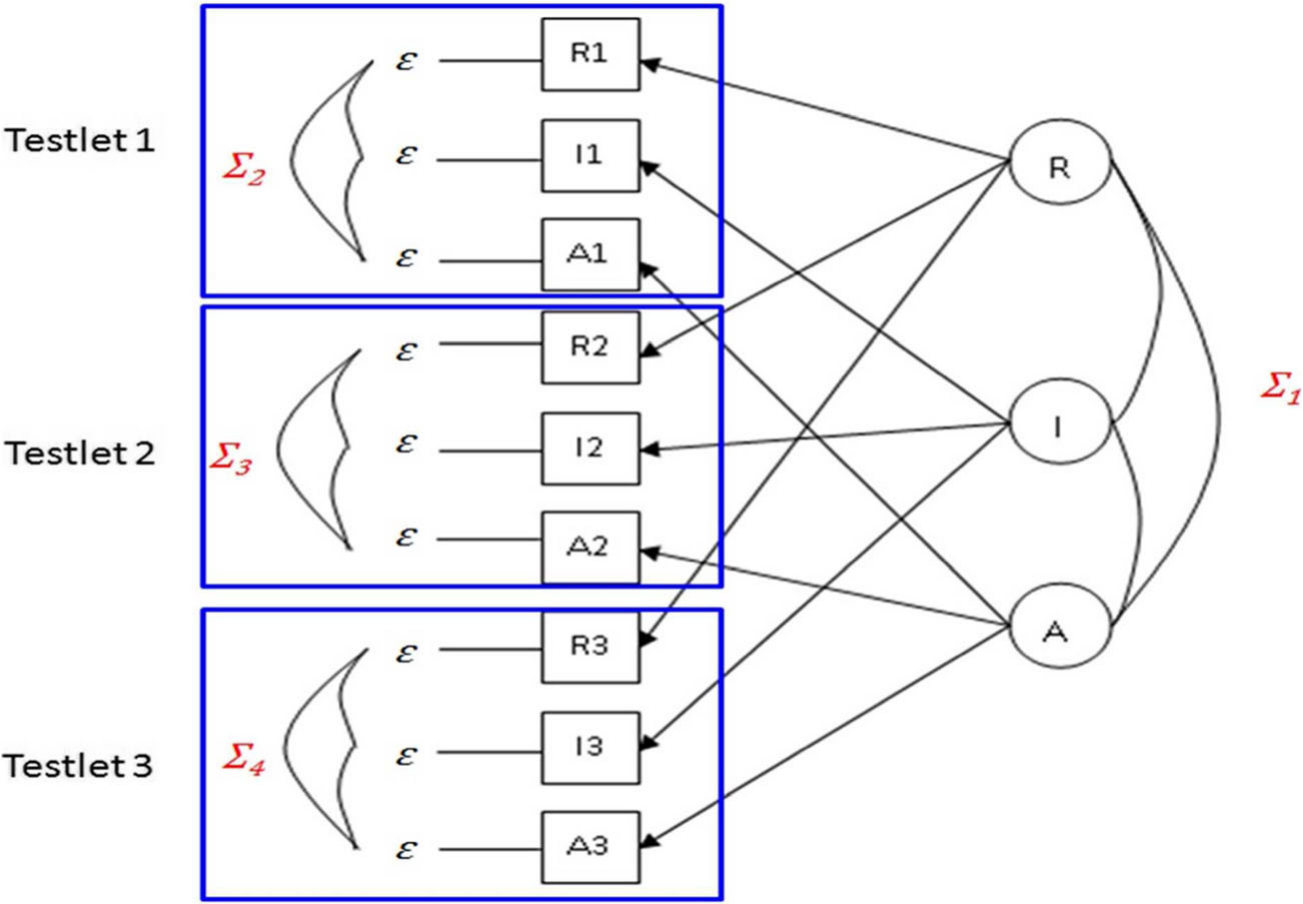
Example of a correlated-traits–correlated-uniqueness model using the Visual Analogue Scale for Rating, Ranking, and Paired-Comparison (VAS-RRP). R = realistic type, I = investigative type, A = artistic type (Holland, 1997).

Figure 2 is an example of the CTCU model when adopting a VAS-RRP to perform CFA. In this example, there are three latent variables (R, I, and A, representing, respectively, the realistic, investigative, and artistic interest types described by Holland, 1997), and the elements in the covariance matrix *Σ1* quantify the correlations between the variables. Each of the latent variables is measured by three items, and *ε* refers to the measurement error of each of them. Since respondents compare three items in each testlet, the three item scores are mutually influenced and correlated. Such correlations or context effects are represented by *Σ*_2_, *Σ*_3_, and *Σ*_4_.

To sum up, on the basis of the data features of the VAS-RRP described above, there are three approaches to analyzing VAS-RRP data: The first one is to use an IRT model or factor analysis to rescale the VAS-RRP data, and then apply statistical procedures to analyze these scaled data. Alternatively, since the VAS-RRP elicits respondents’ fine-grained responses on a line continuum, and the estimators obtained from fine-grained data will be less biased than those derived from Likert scale and ranking (Bollen & Barb, 1981; Krieg, 1999), statistical procedures such as the *t* test, *F* test, and analysis of variance, or descriptive statistics such as the mean, standard deviation, and correlation coefficient of a VAS-RRP, could be applied. Moreover, VAS-RRPs can be used to investigate the relationships among unobservable latent constructs and measured variables, such as through CFA or structural equation modeling (SEM), which may not be eligible for use with ranked data sets.

### Simulation and empirical studies of the VAS-RRP

To demonstrate the advantages of using VAS-RRPs, two simulations were first performed in this study: In Simulation 1 we compared VAS-RRPs with Likert-type scales, and in Simulation 2 we compared VAS-RRPs with ranking, in terms of both parameter recovery and model fit. Next, we also performed an analytical comparison of empirical data from the Situation-Based Career Interest Assessment (SCIA; Sung, Cheng, & Hsueh, 2017; Sung, Cheng, & Wu, 2016) and evaluated the efficacy of the VAS-RRP. Two sets of empirical data obtained using the VAS-RRP and Likert-type scales were then analyzed to demonstrate the differences between these scales.

## Simulation Study 1: VAS-RRPs versus Likert-type scales

Likert-type scales are widely criticized because they use only a small number of response categories for the measurement of latent variables. When the latent variables are fine-grained data, the use of Likert-type scales results in measurement errors. In Simulation 1 we examined the extent to which model fit and parameter recovery are affected by such errors.

### Methods

#### Data of simulation

Two types of simulated data were used that were based on the research objectives of this study: those with and without the context of comparison effects. The first type of data was generated by the CTCU model to simulate a testlet comprising data with the context effects, whereas the second type of data was generated by the correlated-trait model (CT model). Correlated error terms in the CTCU model can represent the context effects (as described in the previous section about the analysis of the VAS-RRP, as well as shown in Fig. 2, which used three latent variables as an example; however, in this simulation we used four latent variables instead of three), meanwhile the error terms of the CT model are not correlated, so the CT model can simulate data that do not exhibit the context effects. While generating the two types of data, we also applied different models for the analysis. There were four latent variables, each of which had either four or eight items. Following the empirical studies of Sung, Cheng, and Wu (2016) and the simulation settings of Brown and Maydeu-Olivares (2012), factor loadings among the latent variables and the items were set to range from 0.60 to 1.20 in each simulation. The coefficient for the correlation among the latent variables was .1 or .3, with the correlation being stronger between two adjacent variables (Holland, 1997). In the CTCU model, the correlation coefficient for the error terms was also set as .1 or .3, with the correlation again being stronger between two adjacent variables.

The CTCU model can generate VAS-RRP data ranging from 0 to 1, whereas the CT model generates continuous data that, through the use of cut points, can be transformed into Likert-scale data (Krieg, 1999; Nyren et al., 1987). The simulated data were generated using Mplus, with the default data based on a standard normal distribution and within a range of [– 3, 3]. In the process of simulating Likert-type scales, we used {– 2, 2}, {– 1, 1}, {– 3, – 1, 1, 3}, and {– 1.5, – 0.5, 0.5, 1.5} as the cut points to represent two types of 3-point Likert-type scales and two types of 5-point Likert-type scales. Note that Likert-type scales with an identical number of response categories that are cut at different values can be used to mimic different category descriptions.

In all simulation scenarios the sample size was 500, and each simulation was run 500 times. For convenience, in the CT model, we use “*x*L*y*I” to represent a model that has *x* latent variables, with each latent variable containing *y* items. In the CTCU model, we use *x*L*y*I to represent a model that has *x* latent variables, with *y* testlets and each latent variable containing *y* items. For the Likert-type scales, 4L8I and 4L4I represent models that have four latent variables, with each latent variable containing eight and four items, respectively. For VAS-RRP scales, 4L8I and 4L4I represent models that have four latent variables with eight or four testlets, each containing four items.

#### Analysis

We used Cronbach’s alpha as well as the model fit indices root mean square error of approximation (RMSEA), standardized root mean square residual (SRMR), comparative fit index (CFI), Tucker–Lewis index (TLI), and *χ*^2^ to assess the model fit to the data, for which the CTCU model was used for the VAS-RRP data and the CT model was used for the Likert-type data. Values of RMSEA < .08, SRMR < .05, CFI > .9, and TLI > .9 (Hooper, Coughlan, & Mullen, 2008), or *χ*^2^/*df* < 3 (Carmines & McIver, 1981) indicate that the model has a good fit. In terms of parameter recovery, the factor loadings, correlations of the latent variables, and correlations of errors were assessed. In addition to Cronbach’s alpha, we established another less biased reliability indicator by measuring the composite reliability of each latent variable (Bagozzi & Yi, 1988; Raykov, 1997; Zimmerman, Zumbo, & Lalonde, 1993). The composite reliability of the *C*th latent variable was calculated as

$$ {\rho}_c=\frac{{\left(\sum \limits_{i=1}^n{\lambda}_i\right)}^2}{{\left(\sum \limits_{i=1}^n{\lambda}_i\right)}^2+\sum \limits_{i=1}^n\operatorname{var}\left({\varepsilon}_i\right)}, $$

where *n* is the number of items of the *C*th latent variable, *λ*_*i*_ is the *i*th factor loading of the *C*th latent variable, and var (*εi*) is the error variance of the items. Bagozzi and Yi pointed out that ρc > 0.6 is required because a higher ρ*c* value indicates that a measured latent variable is more effective.

This study used Mplus 7.0 for further analysis because it provides rapid data simulation. However, this version is not equipped with the principal component method to estimate factor loadings, which is the only method that does not require the covariance matrix to be nonsingular. Therefore, comparisons in Simulation 1 do not include the use of ranking scales. The description of Simulation 2 provides a comparison between a ranking scale and a VAS-RRP.

### Results

#### Model fit

Table 1 lists the mean and standard error (*SE*) values of the Cronbach’s alpha after 500 simulations. Table 1 indicates that the VAS-RRP has higher Cronbach’s alpha than the 3- and 5-point Likert-type scales for the 4L4I and 4L8I models, respectively. In Table 1, we can also find that the values of Cronbach’s alpha are higher for the 4L8I models than for the 4L4I models and higher for the VAS-RRP than for the 3- and 5-point Likert-type scales. Using the software Cocron (Diedenhofen & Musch, 2016) for testing the significance of difference in two coefficients revealed that all the differences of the reliability coefficients between VAS-RRPs and 3-point Likert scales were significant [*χ*^2^(1) ranging from 4.11 to 30.97, all *p*s < .05]; for the differences of the coefficients between the VAS-RRP and the 5-point Likert scales, only those in the {– 3, – 1, 1, 3} conditions were significantly different.

**Table 1:** Reliabilities of different scales

Model	Scale	Cut Points	Cronbach’s Alpha			
			Latent Variable 1 (*SE*)	Latent Variable 2 (*SE*)	Latent Variable 3 (*SE*)	Latent Variable 4 (*SE*)
4L4I	VAS-RRP scale		.713 (.022)	.796 (.015)	.736 (.020)	.811 (.014)
	Likert-type scales	{– 2, 2}	.482 (.046)	.612 (.035)	.518 (.044)	.638 (.031)
		{– 1, 1}	.637 (.027)	.728 (.020)	.663 (.025)	.746 (.019)
		{– 3, – 1,1,3}	.651 (.026)	.745 (.018)	.677 (.025)	.763 (.018)
		{– 1.5, – 0.5, 0.5, 1.5}	.685 (.024)	.770 (.016)	.709 (.022)	.785 (.016)
4L8I	VAS-RRP scale		.820 (.011)	.873 (.008)	.836 (.011)	.882 (.007)
	Likert-type scales	{– 3, – 1, 1,3}	.778 (.014)	.842 (.010)	.796 (.014)	.853 (.009)
		{– 1.5, – 0.5, 0.5, 1.5}	.802 (.012)	.857 (.008)	.817 (.012)	.866 (.008)

Values are mean values after 500 simulations. *SE* = standard error. VAS-RRP = Visual Analogue Scale for Rating, Ranking, and Paired-Comparison. #L = number of latent variables. #I = number of items.

Next, Table 2 lists the mean and standard error (*SE*) values of the model fit indices after 500 simulations. Examination of the VAS-RRP and the different Likert-type scales reveals that all models provided a good fit to the data. There were only minor differences among the fit indices, and increasing the number of response categories did not improve the goodness of fit. We also found that the differences between the Likert-type scales and the VAS-RRP were insignificant on the basis of the model fit indices. The chi-square statistic and fit indices perform well when continuous data are replaced by coarse-grained ordinal scales.

**Table 2:** Model fit indices of the scales

Model	Scale	Cut points	Model Fit Indices					
			RMSEA	SRMR	CFI	TLI	*χ* ^2^	*df*
4L4I	VAS-RRP scale		.008 (.009)	.027 (.003)	.998 (.003)	.999 (.007)	75.56 (12.365)	74
	Likert-type scales	{– 2, 2}	.010 (.009)	.033 (.003)	.987 (.015)	.991 (.026)	103.276 (14.972)	98
		{– 1, 1}	.008 (.009)	.030 (.003)	.995 (.007)	.999 (.013)	99.374 (14.924)	98
		{– 3, – 1, 1, 3}	.007 (.009)	.030 (.003)	.996 (.006)	.999 (.012)	98.783 (14.345)	98
		{– 1.5, – 0.5, 0.5, 1.5}	.008 (.009)	.029 (.003)	.996 (.005)	.998 (.010)	100.686 (14.494)	98
4L8I	VAS-RRP scale		.006 (.006)	.032 (.002)	.998 (.003)	.998 (.005)	421.251 (28.093)	410
	Likert-type scales	{– 3, – 1, 1, 3}	.006 (.006)	.034 (.002)	.996 (.005)	.997 (.007)	468.645 (29.420)	458
		{– 1.5, – 0.5, 0.5, 1.5}	.007 (.006)	.033 (.002)	.996 (.004)	.997 (.007)	471.686 (30.333)	458

Values are mean (*SE*) values after 500 simulations. VAS-RRP = Visual Analogue Scale for Rating, Ranking, and Paired-Comparison. #L = number of latent variables. #I = number of items.

Another way to compare the reliability of Likert-type scales and VAS-RRP is to investigate their composite reliabilities. Table 3 lists the mean values of each factor’s composite reliability after 500 simulations. It shows that the VAS-RRP has higher composite reliability than the 3- and 5-point Likert-type scales for the 4L4I and 4L8I models, respectively. Moreover, the value of composite reliability are higher for Likert-type scales with more response categories and higher for the 4L8I models than for the 4L4I models.

**Table 3:** Composite reliabilities of the different scales

Model		Cut Points	Composite Reliability			
			Latent Variable 1	Latent Variable 2	Latent Variable 3	Latent Variable 4
4L4I	VAS-RRP scale		.718	.799	.741	.815
	Likert-type scales	{– 2, 2}	.495	.620	.529	.646
		{– 1, 1}	.642	.731	.667	.748
		{– 3, – 1, 1, 3}	.657	.749	.683	.767
		{– 1.5, – 0.5, 0.5, 1.5}	.689	.772	.713	.787
4L8I	VAS-RRP scale		.815	.889	.851	.898
	Likert-type scales	{– 3, – 1, 1, 3}	.774	.856	.811	.868
		{– 1.5, – 0.5, 0.5, 1.5}	.798	.871	.811	.881

VAS-RRP = Visual Analogue Scale for Rating, Ranking, and Paired-Comparison. #L = number of latent variables. #I = number of items.

#### Parameter recovery

Table 4 lists the mean and *SE* values of the parameter estimates for the Likert-type scales (with different response categories and varying numbers of response categories) and the VAS-RRPs obtained in the 4L4I model after 500 simulations. First, the results of using a VAS-RRP showed that parameter recovery was ideal in terms of factor loading, correlation of latent variables, and error correlation. Second, the results from using different numbers of categories of Likert-type scales show different estimation bias (i.e., the difference between the mean of estimates and the parameter). Fewer response categories decrease the accuracy of parameter recovery. We can also find that using Likert-type scales cannot obtain estimations for the correlation matrix of error. Finally, comparing the bias of VAS-RRPs and Likert-type scales to the true values, we can find that the biases caused by using VAS-RRPs are smaller than the biases caused by using Likert-type scales. Given that the results were similar for the 4L4I and 4L8I models, Table 4 only lists the results for the former.

**Table 4:** Parameter recoveries obtained by the VAS-RRP scale and Likert scales with different cut points in the 4L4I model

True Value	VAS-RRP Scale	Likert-Type Scale With Cut Points of {– 2, 2}	Likert-Type Scale With Cut Points of{– 1, 1}	Likert-Type Scale With Cut Points of {– 3, – 1, 1, 3}	Likert-Type Scale With Cut Points of {– 1.5, – 0.5, 0.5, 1.5}
Factor loading: (λ1, λ2, λ3, λ4) = (0.65,0.75,0.85,0.95) (λ5, λ6, λ7, λ8) = (1.15,1.05,0.95,0.85) (λ9, λ10, λ11, λ12) = (0.70,0.80,0.90,1.00) (λ13, λ14, λ15, λ16) = (1.20,1.10,1.00,0.90)	Estimates (Mean & *SE*): (.651, .746, .851, .946) (.058, .054, .062, .064) (1.149, 1.047, .948, .847) (.062, .058, .057, .057) (.695, .796, .892, .998) (.057, .059, .064, .063) (1.194, 1.095, .998, .900) (.061, .062, .060, .058)	Estimates (Mean & *SE*): (.109, .135, .168, .196) (.027, .029, .032, .037) (.261, .229, .197, .166) (.030, .028, .027, .027) (.120, .149, .179, .212) (.032, .034, .037, .045) (.275, .244, .212, .183) (.029, .029, .029, .027)	Estimates (Mean & *SE*): (.307, .345, .389, .422) (.034, .031, .034, .034) (.488, .457, .424, .387) (.030, .029, .031, .029) (.326, .367, .404, .439) (.033, .033, .033, .035) (.504, .471, .441, .406) (.030, .030, .031, .030)	Estimates (Mean & *SE*): (.325, .372, .426, .471) (.037, .034, .039, .039) (.571, .521, .474, .423) (.036, .033, .035, .033) (.384, .397, .447, .497) (.035, .037, .038, .040) (.594, .544, .499, .449) (.037, .035, .034, .034)	Estimates (Mean & *SE*): (.598, .671, .752, .819) (.056, .054, .057, .055) (.947, .887, .822, .750) (.051, .048, .051, .051) (.630, .714, .783, .855) (.055, .054, .056, .055) (.975, .916, .855, .787) (.050, .050, .053, .051)
Correlation matrix of latent variables: $$ \left[\begin{array}{cc}\begin{array}{cc}-& .3\\ {}& -\end{array}& \begin{array}{cc}.1& .3\\ {}.3& .1\end{array}\\ {}\begin{array}{cc}& \\ {}& \end{array}& \begin{array}{cc}-& .3\\ {}& -\end{array}\end{array}\right] $$	Estimates: $$ \left[\begin{array}{cc}\begin{array}{cc}-& .299\\ {}.\mathbf{055}& -\end{array}& \begin{array}{cc}.095& .299\\ {}.297& .099\end{array}\\ {}\begin{array}{cc}.\mathbf{065}& .\mathbf{053}\\ {}.\mathbf{054}& .\mathbf{056}\end{array}& \begin{array}{cc}-& .299\\ {}.\mathbf{055}& -\end{array}\end{array}\right] $$	Estimates: $$ \left[\begin{array}{cc}\begin{array}{cc}-& .288\\ {}.\mathbf{088}& -\end{array}& \begin{array}{cc}.088& .282\\ {}.281& .098\end{array}\\ {}\begin{array}{cc}.\mathbf{091}& .\mathbf{089}\\ {}.\mathbf{085}& .\mathbf{071}\end{array}& \begin{array}{cc}-& .283\\ {}.\mathbf{091}& -\end{array}\end{array}\right] $$	Estimates: $$ \left[\begin{array}{cc}\begin{array}{cc}-& .297\\ {}.\mathbf{063}& -\end{array}& \begin{array}{cc}.096& .296\\ {}.295& .098\end{array}\\ {}\begin{array}{cc}.\mathbf{071}& .\mathbf{062}\\ {}.\mathbf{062}& .\mathbf{062}\end{array}& \begin{array}{cc}-& .296\\ {}.\mathbf{064}& -\end{array}\end{array}\right] $$	Estimates: $$ \left[\begin{array}{cc}\begin{array}{cc}-& .299\\ {}.\mathbf{061}& -\end{array}& \begin{array}{cc}.096& .300\\ {}.298& .100\end{array}\\ {}\begin{array}{cc}.\mathbf{069}& .\mathbf{059}\\ {}.\mathbf{060}& .\mathbf{061}\end{array}& \begin{array}{cc}-& .300\\ {}.\mathbf{062}& -\end{array}\end{array}\right] $$	Estimates: $$ \left[\begin{array}{cc}\begin{array}{cc}-& .297\\ {}.\mathbf{059}& -\end{array}& \begin{array}{cc}.096& .295\\ {}.295& .096\end{array}\\ {}\begin{array}{cc}.\mathbf{068}& .\mathbf{056}\\ {}.\mathbf{057}& .\mathbf{058}\end{array}& \begin{array}{cc}-& .296\\ {}.\mathbf{059}& -\end{array}\end{array}\right] $$
Correlation matrix of error: Testlet1 = $$ \left[\begin{array}{cc}\begin{array}{cc}-& .3\\ {}& -\end{array}& \begin{array}{cc}.1& .3\\ {}.3& .1\end{array}\\ {}\begin{array}{cc}& \\ {}& \end{array}& \begin{array}{cc}-& .3\\ {}& -\end{array}\end{array}\right] $$ Testlet2 = $$ \left[\begin{array}{cc}\begin{array}{cc}-& .3\\ {}& -\end{array}& \begin{array}{cc}.1& .3\\ {}.3& .1\end{array}\\ {}\begin{array}{cc}& \\ {}& \end{array}& \begin{array}{cc}-& .3\\ {}& -\end{array}\end{array}\right] $$ Testlet3 = $$ \left[\begin{array}{cc}\begin{array}{cc}-& .3\\ {}& -\end{array}& \begin{array}{cc}.1& .3\\ {}.3& .1\end{array}\\ {}\begin{array}{cc}& \\ {}& \end{array}& \begin{array}{cc}-& .3\\ {}& -\end{array}\end{array}\right] $$ Testlet4 = $$ \left[\begin{array}{cc}\begin{array}{cc}-& .3\\ {}& -\end{array}& \begin{array}{cc}.1& .3\\ {}.3& .1\end{array}\\ {}\begin{array}{cc}& \\ {}& \end{array}& \begin{array}{cc}-& .3\\ {}& -\end{array}\end{array}\right] $$	Estimates: $$ \left[\begin{array}{cc}\begin{array}{cc}-& .299\\ {}.\mathbf{059}& -\end{array}& \begin{array}{cc}.102& .301\\ {}.301& .101\end{array}\\ {}\begin{array}{cc}.\mathbf{051}& .\mathbf{059}\\ {}.\mathbf{059}& .\mathbf{062}\end{array}& \begin{array}{cc}-& .303\\ {}.\mathbf{058}& -\end{array}\end{array}\right] $$ $$ \left[\begin{array}{cc}\begin{array}{cc}-& .299\\ {}.\mathbf{058}& -\end{array}& \begin{array}{cc}.098& .296\\ {}.301& .099\end{array}\\ {}\begin{array}{cc}.\mathbf{054}& .\mathbf{057}\\ {}.\mathbf{058}& .\mathbf{059}\end{array}& \begin{array}{cc}-& .294\\ {}.\mathbf{058}& -\end{array}\end{array}\right] $$ $$ \left[\begin{array}{cc}\begin{array}{cc}-& .304\\ {}.\mathbf{059}& -\end{array}& \begin{array}{cc}.104& .300\\ {}.303& .102\end{array}\\ {}\begin{array}{cc}.\mathbf{055}& .\mathbf{057}\\ {}.\mathbf{058}& .\mathbf{053}\end{array}& \begin{array}{cc}-& .301\\ {}.\mathbf{060}& -\end{array}\end{array}\right] $$ $$ \left[\begin{array}{cc}\begin{array}{cc}-& .301\\ {}.\mathbf{058}& -\end{array}& \begin{array}{cc}.099& .297\\ {}.303& .099\end{array}\\ {}\begin{array}{cc}.\mathbf{062}& .\mathbf{061}\\ {}.\mathbf{054}& .\mathbf{053}\end{array}& \begin{array}{cc}-& .301\\ {}.\mathbf{059}& -\end{array}\end{array}\right] $$				

Values in bold are standard errors. VAS-RRP = Visual Analogue Scale for Rating, Ranking, and Paired-Comparison.

#### Summary

On the basis of several indices, such as the Cronbach’s alpha, parameter recovery, or composite reliability values, this study shows that the measurement errors caused by ordinal scales, such as Likert, clearly affect estimation and reduce the composite reliability. In contrast, VAS-RRPs do not have these problems and help obtain more satisfactory parameter recovery, composite reliability, and Cronbach’s alpha values, especially when compared to Likert scales, which can be as coarse as three points.

## Simulation Study 2: VAS-RRPs versus ranking

Given that ranking scales are ipsative and thus create singular covariance matrices, most statistical techniques are not applicable to such scales. In Simulation 2, we used exploratory factor analysis (EFA) with the principal component method (Dunlap & Cornwell, 1994; Loo, 1999) to estimate parameters, and then we compare the model fit and parameter recovery between the VAS-RRP and the ranking.

### Methods

#### Simulation data

This study randomly selected one of the 4L4I and 4L8I datasets of the VAS-RRP generated by the CTCU model in Simulation Study 1. The numeric values of each item on a VAS-RRP can be transformed into ranking data through their orders on the VAS-RRP continuum. Since the results of the two datasets were similar, to save space, this section only presents the analysis and results for dataset 4L4I.

#### Analysis

In Simulation 2 we used SPSS to apply EFA in order to compare differences in model fit and parameter recovery for the VAS-RRP and the ranking data. We compared the model fit of the scales based on the proportion of variance explained (PVE), Cronbach’s alpha, and factor structure. Estimates of parameter recovery for the factor loadings and the correlation of the latent variables were also evaluated.

### Results

#### Factor structure and parameter recovery

Table 5 compares the VAS-RRP and the ranking scale in terms of factor structure, which only includes absolute factor loading values of > .3. The VAS-RRP obtained a factor structure similar to the real data. Although the ranking scale still showed four latent variables, the factor structure was very different from the original one. Moreover, the factor structure obtained from the ranking scale changed from a simple to a complex structure, which means that some of the observed variables were now affected by multiple latent variables rather than only one. In contrast, using a VAS-RRP not only obtained a four-factor structure, but also retained a simple structure.

**Table 5:** Factor structures of different scales

	True Value				Ranking Scale				VAS-RRP			
	Component				Component				Component			
	Factor 1	Factor 2	Factor 3	Factor 4	Factor 1	Factor 2	Factor 3	Factor 4	Factor 1	Factor 2	Factor 3	Factor 4
V11	.65						.662					.749
V21		1.15				.823				.783		
V31			.70		.748						.722	
V41				1.20		– .416	– .377		.828			
V12	.75					– .358	.839					.730
V22		1.05				.842				.759		
V32			.80		.741						.783	
V41				1.10	– .372	– .342	– .356		.813			
V13	.85							– .777				.711
V23		.95				.726				.832		
V33			.90		.761						.775	
V43				1.00		– .559		.381	.767			
V14	.95						.532	– .447				.775
V24		.85					.321	.651		.697		
V34			1.00		.734						.771	
V44				.90			– .687		.784			

Comparing the factor loading values of the two scales listed in Table 5, we can find that the factor loadings of the VAS-RRP were generally more desirable than those obtained for the ranking scale. This is due to the factor loading estimates being closer to the actual values. When the ranking scale was adopted, some of the factor loading estimates showed negative values and were far from the actual values of 0.6 to 1.2.

Table 6 compares the two scales in terms of the correlation of the latent variables. The VAS-RRP demonstrated better results than the ranking scale. The ranking scale showed unsatisfactory parameter recovery and mistakenly calculated a positive correlation between some of the variables as a negative one, which severely impacted the inference and interpretation of the latent variables.

**Table 6:** Parameter recovery for the different scales in terms of correlation of the latent variables

Latent Trait	True Value			VAS-RRP			Ranking Scale		
	2	3	4	2	3	4	2	3	4
1	.300	.100	.300	.220	.224	.125	.204	.206	– .128
2		.300	.100		.320	.274		.346	.113
3			.300			.275			– .230

VAS-RRP = Visual Analogue Scale for Rating, Ranking, and Paired-Comparison.

#### Cronbach’s alpha

Table 7 lists the Cronbach’s alpha values of the latent variables for the two scales, which were higher for the VAS-RRP than for the ranking scale. Using Cocron (Diedenhofen & Musch, 2016) to test the two Cronbach’s alpha coefficients of each factor in the two scales showed that all alpha coefficients were significantly different [*χ*^2^(1) = 10.02, 9.35, 8.35, and 13.06 for the four factors, respectively; all *p*s < .01]. Table 7 also lists the PVEs of these two scales; the PVE of the VAS-RRP was slightly higher than that of the ranking.

**Table 7:** Reliability and proportions of variance explained (PVEs) for the different scales

	Cronbach’s Alpha				PVE
Scale	Factor 1	Factor 2	Factor 3	Factor 4	
Ranking	.636	.696	.689	.733	58.85%
VAS-RRP	.731	.773	.764	.811	59.66%

VAS-RRP = Visual Analogue Scale for Rating, Ranking, and Paired-Comparison.

#### Summary

Our findings indicate that the ipsative data produced by ranking has resulted in limitations on statistical analysis, such as unsatisfactory parameter recovery for factor loadings and correlation of latent variables, or incorrect estimation of the correlation of latent variables. Our results indicate that the use of a VAS-RRP can avoid these unwanted effects.

## Empirical Study 1: Comparing the VAS-RRP and Likert scales for career interest assessment

In this study, the model fit, reliability, PVE, composite reliability, leniency biases, and covariance matrices from the participants’ actual responses were compared through empirically collected data from the VAS-RRP and Likert scales.

### Methods

#### Assessment tool and data collection

**Fig. 3:**
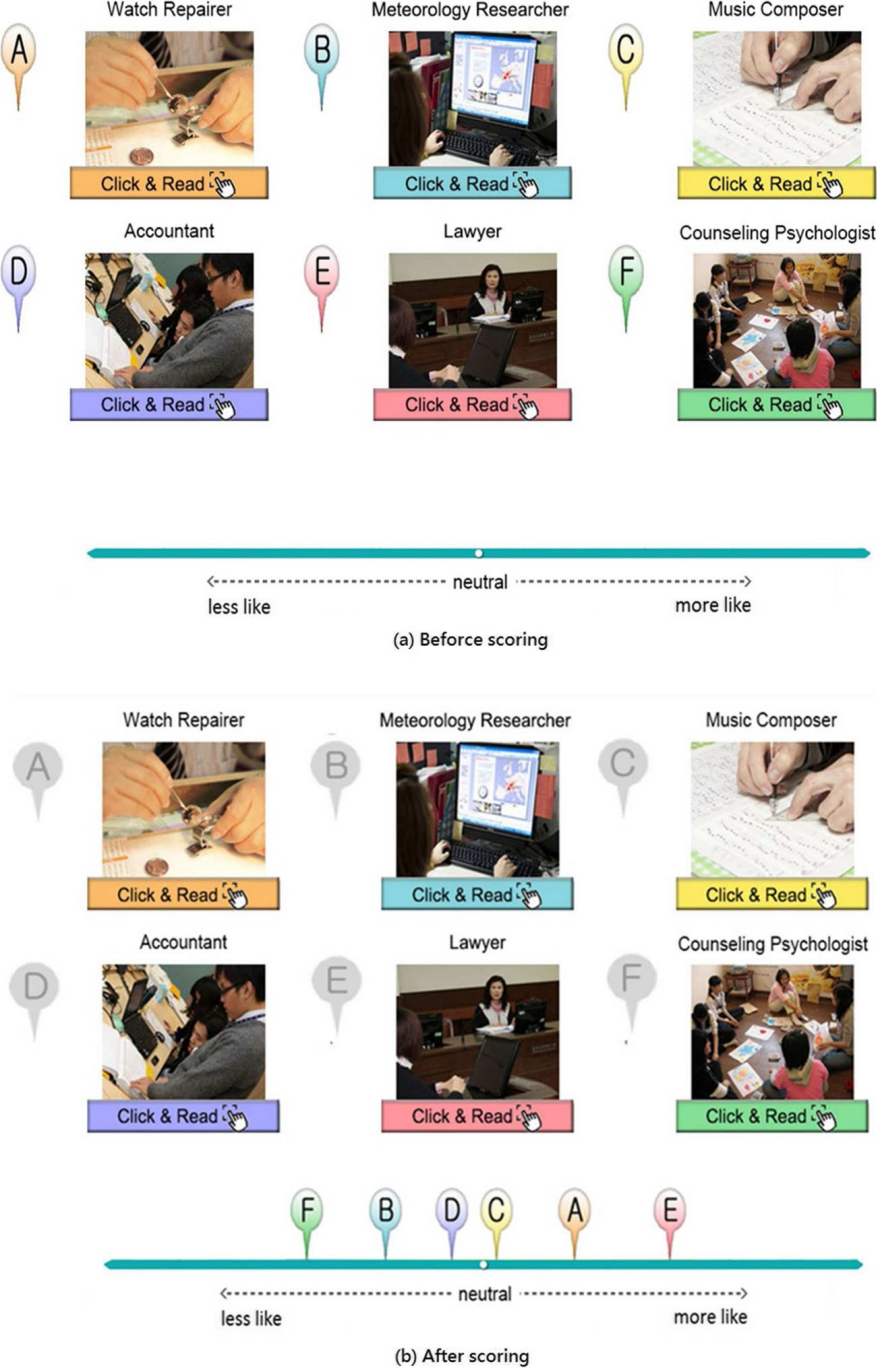
Example of a Situation-Based Career-Interest Assessment testlet.

The Situation-Based Career Interest Assessment (SCIA; Sung, Cheng, & Hsueh, 2017; Sung et al., 2016) is a situation-based, computerized interest test that is based on the theory of career choice reported by Holland (1997). With the help of information and multimedia technology, the SCIA was designed to assist students in grades 7 to 9 with their career choices. According to Holland, career interests can be divided into six different types: realistic, investigative, artistic, social, enterprising, and conventional. A simplified version of the SCIA contains 54 items, comprising nine testlets with items from each of the six career types was used in this study. The testlets were provided to respondents one at a time during the assessment, with the items related to the six interest types randomly placed on a computer screen (see the items A to F in Fig. 3a). Considering that junior high school students may not be familiar with the titles of vocations, SCIA provided photos along with descriptions under each vocation’s title, and allowed students to click the vocation’s icon to learn more about it. SCIA used a VAS-RRP with midpoints labeled *neutral*. Indications made toward the right side of the scale refer to increasing preference and those toward the left imply increasing aversion. After comparing the items, the respondent could move the icons labeled from A to F to any point along the scale that they considered to be suitable, with the positions used for subsequent scoring (Fig. 3b). Students had one practice testlet before they answered the formal testlets.

Another data set was also obtained by using a Likert-type scale, for comparison. The Likert-type scale asked the same respondents to rate their preference or aversion for each of 54 items displayed on a computer screen by responding on the following 5-point scale: *very unfavorable*, *unfavorable*, *neutral*, *favorable*, and *very favorable*. A counterbalanced design was used in which about half of the respondents performed their ratings using the VAS-RRP before proceeding to the 5-point Likert-type scale, whereas the other respondents used the Likert-type scale first. It was not necessary to collect ranking data since they could be obtained simply by transforming the VAS-RRP data. This study collected 1,749 valid samples of 9th grade students in junior high schools (average age 15.2), among them 933 were males and 816 were females. All the students’ parents approved of their children’s participation in the research before data collection commenced.

#### Analysis

We first analyzed the model fit. The CTCU and CT models were used for the VAS-RRP and Likert-type data, respectively. Furthermore, the reliability, PVE, and composite reliability were also analyzed. We also compared the three scales in terms of their leniency biases, and differences in covariance matrices. The leniency bias refers to whether bias or errors existed in the respondents’ ratings and rankings, and this was calculated by comparing the mean and median values for the six interest types—a greater difference indicates a larger leniency bias (Chiu & Alliger, 1990) and that the respondents are more likely to provide overstated or understated ratings. A comparison of covariance matrices helps in examining whether the additional comparison procedure in the VAS-RRP affects the covariance matrix in a way similar to what happens when a rating scale is applied. Finally, the amounts of time participants required in order to complete the scales were also compared.

### Results

#### Model fit, reliability, and PVE

Table 8 lists the model fit indices when using the Likert-type scale and the VAS-RRP. The model fit of the Likert-type scale was generally similar to that of the VAS-RRP. These outcomes are consistent with the simulation results obtained in this study, indicating that the Likert-type scale and the VAS-RRP showed only minor differences in fit indices.

**Table 8:** Model fit indices of different scales

	Likert-Type (CT Model)	VAS-RRP (CTCU Model)
RMSEA	.079	.080
CFI	.923	.936
TLI	.919	.925
SRMR	.095	.099

VAS-RRP = Visual Analogue Scale for Rating, Ranking, and Paired-Comparison. CT = correlated trait. CTCU = correlated traits–correlated uniqueness

Table 9 lists the Cronbach’s alpha, composite reliability, and PVE for each of the six interest types. Cronbach’s alpha was highest for the VAS-RRP and lowest for the ranking scale, using Cocron (Diedenhofen & Musch, 2016) to test the differences of coefficients, the subscales I, A, E, and C [*χ*^2^(1) = 4.17, 9.48, 12.98, 6.52, respectively] of the VAS-RRP were significantly higher than those of Likert scales. The differences of the reliability coefficients of the VAS-RRP and ranking were all significantly different [*χ*^2^(1) ranges from 63.45 to 441.98, all *p*s < .01]. The composite reliability was also higher for the VAS-RRP. Moreover, the VAS-RRP had the highest PVE at 55.75%, followed by the Likert-type scale at 52.96%, whereas the ranking scale showed the smallest PVE at 44.18%.

**Table 9:** Reliabilities and proportions of variance explained (PVEs) for the different scales

Latent Trait	Cronbach’s Alpha			Composite Reliability	
	VAS-RRP Scale	Likert-Type Scale	Ranking Scale	VAS-RRP Scale	Likert-Type Scale
R	.918	.912	.879	.997	.910
I	.900	.891	.807	.997	.926
A	.856	.836	.795	.997	.924
S	.847	.836	.737	.998	.929
E	.854	.830	.657	.997	.898
C	.834	.812	.673	.998	.917
PVE	55.75%	52.96%	44.18%		

R, I, A, S, E, and C refer to the following interest types described by Holland (1997): realistic, investigative, artistic, social, enterprising, and conventional, respectively. VAS-RRP = Visual Analogue Scale for Rating, Ranking, and Paired-Comparison.

#### Leniency bias

The leniency bias is calculated by subtracting the median from the mean. A value < 0 means that participants’ ratings tend to concentrate on the right side of the scale, showing increasing preference, whereas a value > 0 indicates that participants’ ratings incline toward the left of the scale, indicating increasing aversion. Given that the three scales had different ranges (i.e., 0–1, 1–5, and 1–6 for the VAS-RRP, 5-point Likert, and ranking scales, respectively), prior transformations were required so that all values fell within the range of 0–1 to make direct comparison possible; this was achieved by dividing the Likert data by 5 and the ranking data by 6. Table 10 compares the leniency bias values of the three scales. Overall the leniency bias of the VAS-RRP was close to 0, indicating fewer extreme responses (e.g., “very favorable” or “very unfavorable”) with this scale.

**Table 10:** Leniency bias values for the different scales

	R	I	A	S	E	C
VAS-RRP scale	.003	.005	.004	.004	.003	– .003
Likert-type scale	.012	.004	– .010	– .012	.007	– .014
Ranking scale	– .029	– .022	.000	.006	– .015	– .014

VAS-RRP = Visual Analogue Scale for Rating, Ranking, and Paired-Comparison. R, I, A, S, E, and C refer to the following interest types described by Holland (1997): realistic, investigative, artistic, social, enterprising, and conventional, respectively.

#### Covariance matrix

Ranking scales are ipsative; hence, they create a covariance matrix whose columns and rows always sum to zero. This resulting singular matrix makes it impossible to apply other methods for subsequent analysis. Table 11 presents the covariance matrices of the VAS-RRP and the ranking scale for Testlet 2 of the SCIA. The obtained data indicate that the ranking scale does indeed create the above-mentioned problems, whereas the data with similar context effects obtained when using the VAS-RRP were free of such problems. These findings were also obtained for the other testlets in the SCIA (data not shown).

**Table 11:** Covariance matrices of different scales

	Ranking Scale						VAS-RRP					
	R	I	A	S	E	C	R	I	A	S	E	C
R	.09	.00	– .03	– .03	– .02	– .02	.05	.01	.00	.00	.01	.00
I	.00	.07	– .01	– .02	– .02	– .02	.01	.05	.01	.00	.01	.00
A	– .03	– .01	.09	.00	– .03	– .02	.00	.01	.05	.01	.00	.00
S	– .03	– .02	.00	.06	– .01	.00	.00	.00	.01	.03	.00	.00
E	– .02	– .02	– .03	– .01	.07	.00	.01	.01	.00	.00	.04	.02
C	– .02	– .02	– .02	.00	.00	.06	.00	.00	.00	.00	.02	.03

Values have been rounded to two decimal places. VAS-RRP = Visual Analogue Scale for Rating, Ranking, and Paired-Comparison. R, I, A, S, E, and C refer to the following interest types described by Holland (1997): realistic, investigative, artistic, social, enterprising, and conventional, respectively.

#### Time to completion

The participants took 919.65 s (*SD* = 229.95) on average to complete the VAS-PRP and 461.18 s (*SD* = 119.43) on average to complete the Likert scale. The paired *t* test revealed a significant difference [*t*(1748) = – 86.23, *p* < .01] between the amounts of time spent on the two scales.

#### Summary

The empirical data produced results similar to those of the two simulation studies. Using the VAS-RRP produced higher reliability and PVE. Moreover, with the comparison function of items in the same testlet, the VAS-RRP also reduced leniency bias, which maybe resulted from the longer time engaged with the scale. Despite the similar function of ranking and paired comparison, data collected from the VAS-RRP were not ipsative as produced by ranking and paired comparison, and could thus keep the appropriate property of covariance matrices, which enabled further statistical analyses such as factor analysis.

## Empirical Study 2: Comparing the VAS and VAS-RRP for career interest assessment

This study compared the reliability, leniency biases, and time latency from the participants’ responses for the VAS and VAS-RRP.

### Method

#### Assessment tool and data collection

The SCIA, which was introduced in Empirical Study 1, was used in this study. Another data set was also obtained using a VAS, for comparison. Instead of using a testlet for comparing and ranking items, the VAS version of SCIA individually and randomly presented the 54 items to each participant. In this study we collected two data sets from two groups of participants: The first data set included 246 valid samples of 9th grade in junior high schools (average age 14.9; 132 females and 114 males) for the SCIA VAS; the second included 251 9th graders (average 15.1; 118 females and 133 males) for the SCIA VAS-RRP. All of the students’ parents approved of their children’s participation in the research before data collection began.

#### Analysis

The analyses of reliability, leniency biases, and time latency were identical to the methods used in Empirical Study 1.

### Results

Table 12 lists the Cronbach’s alphas and leniency biases of the two scales. The VAS showed slightly higher reliability than the VAS-RRP; according to the Cocron test results (Diedenhofen & Musch, 2016), the reliability index difference of subscales E and C (Cronbach’s alpha = .956 and .960, respectively) of the VAS was significantly higher than that for the subscales E and C (Cronbach’s alpha = .923 and .928, respectively) of the VAS-RRP [*χ*^2^(1) = 18.36 and 16.78, *p* < .001]. Moreover, overall the leniency bias of the VAS-RRP was close to 0 and much smaller than that of the VAS, indicating fewer extreme responses (e.g., “very favorable” or “very unfavorable”) in the VAS-RRP than in the VAS. The analysis of the amounts of time taken to complete the two scales showed that participants took less time on the VAS (*M* = 963.45 s, *SD* = 311.97) than on the VAS-RRP (*M* = 1,073.55 s, *SD* = 292.33) [*t*(495) = – 4.06, *p* < .01].

**Table 12:** Reliability coefficients of Cronbach’s alpha and leniency bias for the VAS and the VAS-RRP

Type	Cronbach’s *α*		Leniency	
	VAS (*N* = 246)	VAS-RRP (*N* = 251)	VAS (*N* = 246)	VAS-RRP (*N* = 251)
R	.944	.939	.0219	– .0026
I	.955	.941	.0212	– .0034
A	.940	.938	.0012	.0039
S	.945	.936	.0160	.0030
E	.956	.923	.0105	– .0001
C	.960	.928	.0120	– .0020

VAS = visual analogue scale; VAS-RRP = Visual Analogue Scale for Rating, Ranking, and Paired-Comparison; R, I, A, S, E, and C refer to the following interest types described by Holland (1997): realistic, investigative, artistic, social, enterprising, and conventional, respectively.

## Constructing the VAS-RRPs

**Fig. 4:**
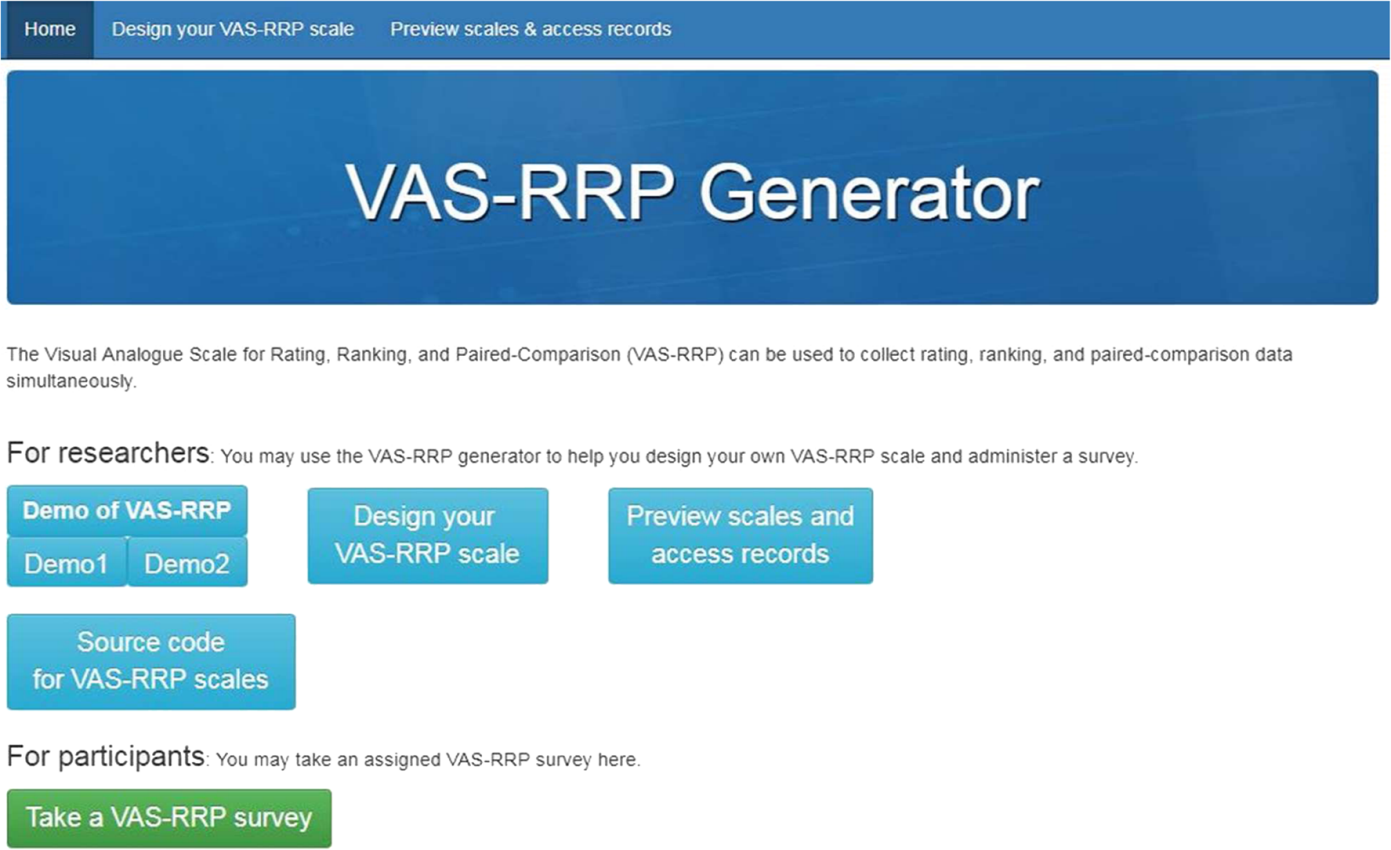
First page of VAS-RRP Generator.

To assist researchers and practitioners with constructing their own VAS-RRPs with ease, we have developed the VAS-RRP Generator (www.vasrrp.net). The generator is an authoring tool that researchers and practitioners can use to easily construct their own VAS-RRPs, administer a survey and collect data for further analysis (Fig. 4). The VAS-RRP Generator uses both the drop-down menu and a template file (with the Excel format) as an authoring tool for researchers to design their own VAS-RRP. Below we explain how to use the VAS-RRP Generator to construct scales and access their data.

- **Step 1: Determine the number of items in each testlet** The number of items in each testlet will determine the task for the participants and the data collected. As we have mentioned, the VAS-RRP can be used for rating, ranking, and paired comparisons. If there is only one item in each testlet, then the VAS-RRP is identical to a regular VAS, and the task that participants need to execute is simply rating the item on the line continuum. If there are two items in each testlet, then the participants need to execute the paired comparison task through dragging and dropping the items onto the line continuum. If there are three or more items in each testlet, then the participants need to execute the ranking task through dragging and dropping the items onto the line continuum. Researchers may determine the items in each testlet according to their theoretical constructs or their practical needs. For example, researchers may need the two-item paired-comparison format because they need to construct a scale for the bipolar personality traits (e.g., introvert vs. extravert); or they may need a six-item ranking format for the hexagonal model of Holland’s (1997) interest types; or they may want to compare the same feature of four brands of cars. Generally we recommend that the items in each testlet cover all the dimensions/factors of a certain psychological construct. For example, if there are six dimensions of a work-value theory, then six items representing the six dimensions are recommended to be included in the same testlet. The first item represents the first dimension/factor of the construct to be investigated, the second item represents the second dimension/factor of the construct, and so on. The positions of those items will be randomly presented. Researchers can use the drop-down menu to determine their items in each testlet.
- **Step 2: Determine the question in each testlet** Each testlet should contain one question that asks participants to express their feelings, attitudes, or opinions, such as “How would you like the vacations below?,” “Which brand of car do you like the most?,” or “In your work environment, which one below would you value most?.” On the basis of the purposes and needs of the researchers, usually the score of the items representing the same dimensions in different testlets can be summed up for a subtotal score for the subscale of the dimension; or the scores of different dimension/subscale can summed up for the total score of the whole scale. Therefore, the same question may be applied to different testlets so long as the items differ. Questions can also be altered across different testlets to increase the diversity of expression (such as replacing the question “In your work environment, which one below would you value most?” with “Which company offer below attracts you most?”) However, researchers have to ensure that different questions across testlets elicit responses belonging to the same target variable.
- **Step 3: Determine the content of items in each testlet** Each of the items in a testlet should be presented as verbal statements (e.g., “watch repairer” as a kind of vacation) or as graphics/pictures (e.g., the pictures showing the working environment of a watch repairer).
- **Step 4: Determine the anchors for the scale in each testlet** On the right and left ends of the line continuum scale, there are two anchors for guiding participants’ expressions of their levels of feeling, attitudes, or opinions. The two anchors are usually bipolar verbs (e.g., agree, disagree) or adjectives (e.g., pleasant, unpleasant), which represent two increasingly opposite levels of attitudes, thoughts, or feelings. Usually the same anchors can be applied to different items and testlets.
- **Step 5: Determine the number of testlets in the whole scale** Usually a scale will include several testlets, based on how many items would be enough to measure the psychological construct, opinions, or attitudes with acceptable reliability and validity.

**Fig. 5:**
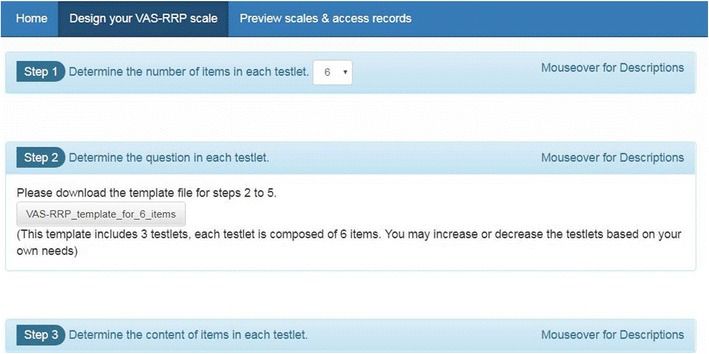
Snapshot of the procedure for the Design_your_VAS-RRP_scale functionality.

**Fig. 6:**
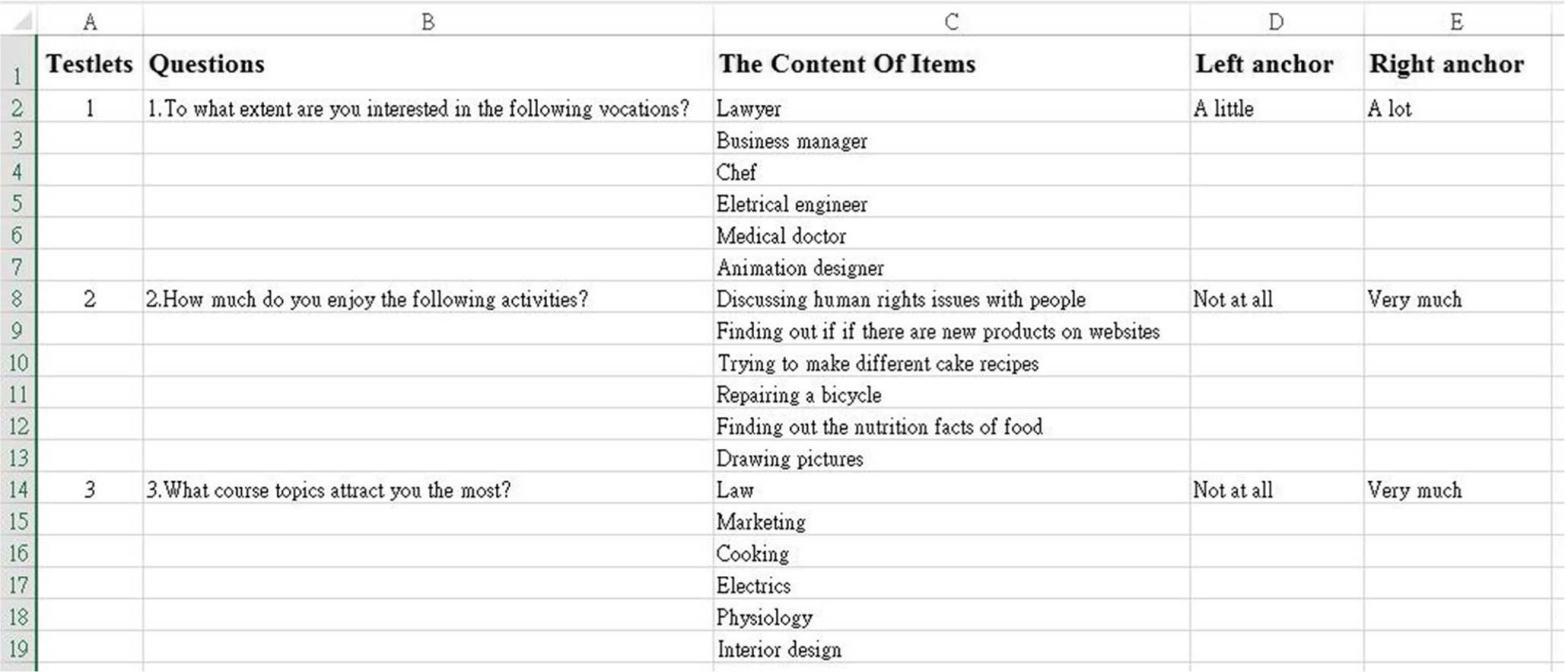
Snapshot of the VAS-RRP template file for three testlets with six items.

For researchers to complete Steps 2–5, they may download the Excel template (Figs. 5 and 6) from the website and revise the content of each item and the anchors for each testlet. The process can be repeated to create the number of testlets desired by the researchers. After the Excel template is completed, it may be uploaded to the website and the system will automatically construct and present the user-designed scale.

**Fig. 7:**
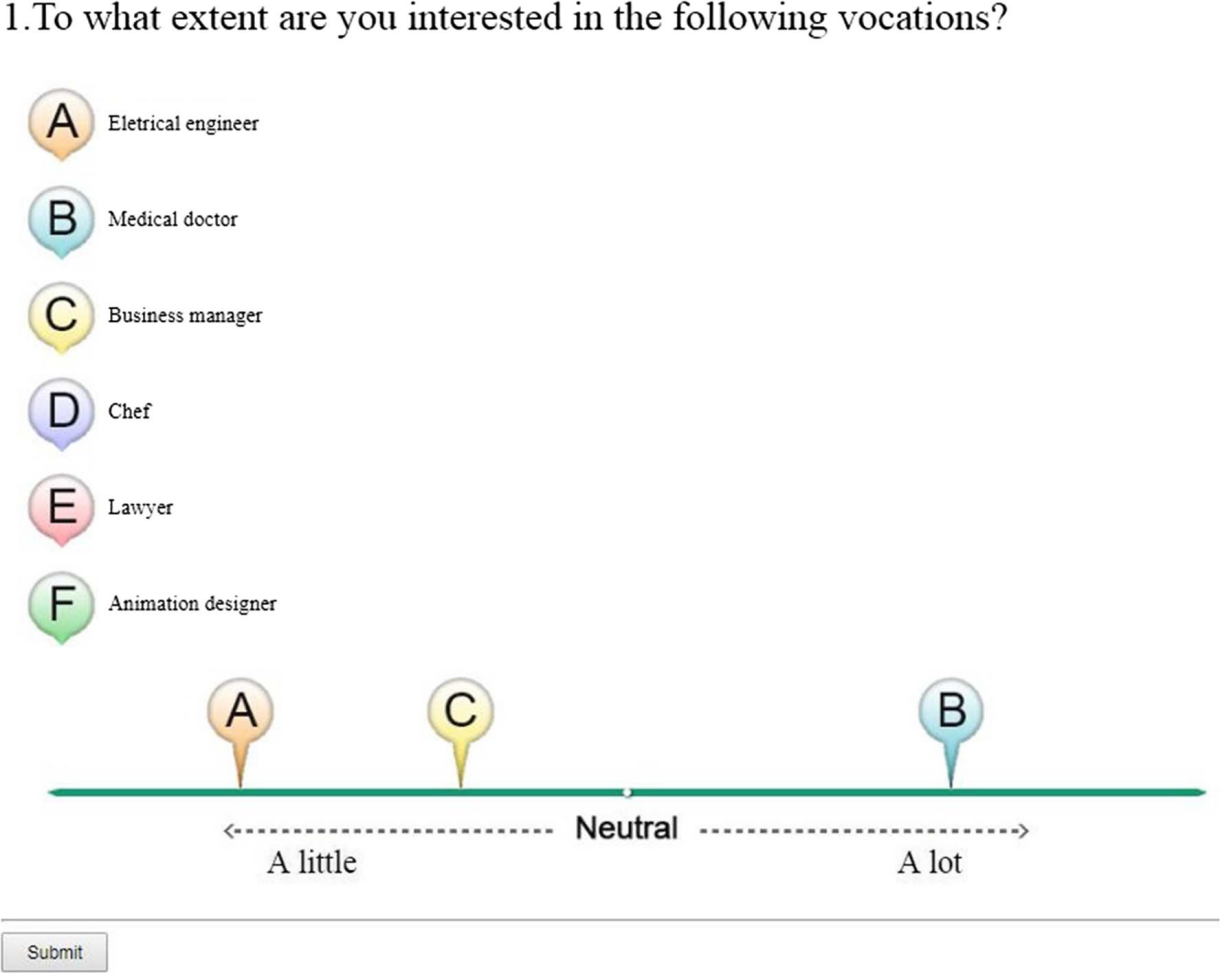
Snapshot of a Take_a_VAS-RRP_survey testlet.

- **Step 6: Preview the scale** Using the “Preview and Record” button, researchers may test the scale they have constructed in advance to see whether it can fulfill their needs. They can revise the Excel template if they need to revise the scale. Researchers may also change the style of the scale, such as the length, width, and colors of the line continuum or the shape and colors of the icons, by using the “Chang Style” function. The testing data, which are the positions of each item on the line continuum, will be converted to values ranging from 0 to 1 as the score of each item, and then will be exported to an Excel output file for the researchers’ reference.
- **Step 7: Administer the scale** After the researchers confirm the number and content of items in each testlet, as well as the number of testlets in the whole scale, they may submit the scale for administration. Researchers need to create a file name and instruction for the scale, which will be used for identification and explanation of the scale. They also need to create a password with which their participants will be allowed to access the scale. After these procedure, researchers can inform their study’s participants of the URL (i.e., www.vasrrp.net), the name of the scale, and the password for the scale. Then, their participants may log onto the website and press the “Take a VAS-RRP survey” button to respond to the assigned scale. The responses of each participant, which are the positions of each item on the line continuum (Fig. 7), will be converted to values ranging from 0 to 1 as the score of each item and will then be exported to an Excel output file.

After the administration of their survey, through the “Preview and Record” button on the website, using the created file name of the scale and the password for accessing the records, researchers may download the aggregated data of all the participants’ responses in the exported Excel file. In the file, each row includes a participant’s number, the date and time of taking the survey, and their scores of each item in each testlet, which are arranged in the order tetslet1_item1, testlet1_item2 . . . testlet2_item1, testlet2_item2, and so on.

## General discussion

When encountering the issues surrounding the limitations of Likert-type scales, such as response styles and ordinal measurement data, researchers may adopt four approaches (Brown, 2014; Spooren, Mortelmans & Thijssen, 2012; Tabachnick & Fidell, 2001): The first one is ignoring the problems and treating all ordinal data as interval. The second approach is changing the format of the scales, such as choosing scales with comparison functions, such as ranking, to overcome the response-style biases caused by using Likert, but ignoring the problems of ipsative measures (e.g., Kolb, 2005; McCloy et al., 1999b). The third method is using a VAS to obtain fine-grained measurements to avoid the measurement errors of Likert-type scales, but accepting that the data may still contain response-style biases and encounter problems with absolute judgments (Wewers & Lowe, 1990). The fourth approach is developing mathematical models coupled with paired comparison or ranking to overcome the limitations of ipsative data (e.g., Brady, 1989; Brown & Maydeu-Olivares, 2011, 2013; Chan & Bentler, 1998; Jackson & Alwin, 1980), while enduring the added burden such methods entail. Despite their possible contributions, all of these proposed methods introduce new problems along with their solutions.

The VAS-RRP proposed in this study offers a fifth approach for overcoming the difficulties researchers encounter. In addition to the convenience of freeing researchers/practitioners from being concerned with the issues of the optimal number of points (categories) on the Likert-type scale (Alwin, 1992; Cox, 1980; McKelvie, 1978; Preston & Colman, 2000), the VAS-RRP’s finer-grained measurements improved the psychometrical properties of Likert-type scales, and the Cronbach’s alpha, parameter recovery, and the composite reliability values were all substantially enhanced. These findings provide more converging evidence for previous claims (e.g., Babakus et al., 1987; Krieg, 1999) that coarse-grained and ordinal data, such as that produced by Likert-type scales, were more prone to measurement errors and reduced reliability. However, our expectation that a fine-grained scale, such as a VAS-RRP, would have superior reliability was not completely fulfilled. First, in our simulation studies, the reliability of 4L8I was similar to the reliability of the VAS-RRP and was better than that of 4L4L, which indicates that a larger number of items in a scale may alleviate the problem of discrete response bias. Secondly, the simulation results revealed that the reliability of the VAS-RRP was not significantly higher than the 5-point Likert scale; our empirical study also found that the VAS-RRP only significantly outperformed the Likert scale in two thirds of the sub-scales. These findings provide support for the previous findings that fine-grained scales were not necessarily superior to coarse-grained scale in terms of reliability (Kuhlmann et al., 2017; McKelvie, 1978). More simulated and empirical studies with different types of designs are needed to clarify these mixed findings.

Another feature of the VAS-RRP is that instead of using a single item for judgment in each scale as in a traditional VAS, the VAS-RRP employed a multi-item (i.e., a testlet) format along with each scale. This innovation not only made the traditional VAS become a special case of the VAS-RRP, but also brought about several advantages. Firstly, the multi-item VAS-RRP enabled more possible types of scaling, such as ranking and paired comparison, when compared to the traditional VAS, which allows only for rating. The multi-item format also allowed respondents to make relative judgments instead of absolute judgments, which should reduce measurement error (Laming, 2004; Nunnally, 1967). Our empirical study showed that the multi-item testlet format of the VAS-RRP effectively reduced response-style bias when compared with a similar Likert-type scale by enabling relative judgments of career interests. This functionality is especially beneficial for the psychological tests focusing on revealing the within-individual differences of dimensions of traits, such as styles, interests, or values. This advantage was illustrated by the fact that the multi-item VAS-RRP helped reduce leniency bias in our two empirical studies. As compared with either Likert scales or VASs, which were not able to curtail participants’ response styles, the VAS-RRP elicited less leniency-bias, which may have resulted from participants spending more time judging their relative preferences for those items shown on the line continuum. However, it is noteworthy that the longer response latencies for the VAS-RRP than for the VAS may also represent a disadvantage, since previous studies using paired-comparison formats have been criticized for being too time-consuming (e.g., McCloy, et al., 1999a). Since the comparison of response latencies for the VAS and VAS-RRP resulted from our second empirical study, which was a between-subjects design, more rigorous designs, such as a within-subjects design along with think-aloud protocols regarding participants’ mental processes of comparison, would help uncover more facts about the different mental operations at work while taking a VAS-RRP or VAS.

Second, integrating the multi-item testlet format with the fine-grained measurements of VAS allowed quantitative comparisons of targeted traits in ranking and paired-comparison tasks, for which only qualitative comparisons were allowed traditionally. Furthermore, the raw data for comparisons produced by the VAS-RRP could be more meaningful than Likert scale, ranking, or paired-comparison scores when calculating regular statistics such as means, standard deviations, correlations, and covariance matrices, with no concern for the problems associated with a same summed-total scale score across participants and singular covariance matrices produced by traditional ranking and paired comparison tasks. In our simulation and empirical studies, the raw data produced by traditional ipsative methods, such as rankings and paired comparisons, clearly demonstrated the limitations mentioned above. However, such disadvantages were alleviated by the VAS-RRP, as more satisfactory covariance matrices and parameter recovery for factor loadings, correlations of the latent variables, and estimations of the correlations of latent variables were found in VAS-RRP data.

Third, despite their ipsative nature, coupled with appropriate models such as CTCU, the VAS-RRP data were appropriate for model fitting and theory testing. This resolved the limitations of traditional ranking and paired comparisons, which could not produce data eligible for model fitting. Our simulation and empirical studies also demonstrated satisfactory parameter recovery using the VAS-RRP. When fitting VAS-RRP data with the CTCU model to explore or confirm theories, they can provide higher reliability than ranking data by modeling the relationships of the latent variables, measurement error, and the context effects in the same testlet, simultaneously. Although our findings supported the usefulness of the VAS-RRP data for overcoming the limitations of using ranking and paired-comparison tasks in model fitting, the model fit indices of the VAS-RRP did not outperform those from Likert-type scales in the present studies. More research with different psychological traits and different VAS-RRP designs will be needed to explore the capability of VAS-RRP designs to enhance the construct validity of scales. Furthermore, as the VAS-RRP was presented in a testlet format, the drag-and-drop operation of items and the line continuum with a neutral point represents a special arrangement different from the traditional VAS. Whether this affects the generalizability of our present research results to other VAS formats will be worthy of more consideration in future research.

On the basis of their multiple functions, ease of use, and eligibility for various statistical analyses, VAS-RRPs can be easily applied to existing assessment tools and may subsequently overcome some of the limitations posed by Likert-type, visual analogue, or ranking scales. For example, the Minnesota Importance Questionnaire (Gay et al., 1971) and the Kolb Learning Style Inventory (Kolb, 2005) are both ipsative measures; however, VAS-RRP data can be obtained by slightly changing the methods used by respondents to provide answers/indications. Another example is the Gordon Personal Profile Inventory (GPPI; Gordon, 1993), in which the scoring is performed by partial ranking: Respondents have to select two items out of four (i.e., the most like me and the least like me), and a considerable amount of item information is lost. Such information loss would not occur if we used the VAS-RRP to produce the ranking data in the GPPI. Furthermore, a VAS-RRP can also work in place of a Likert-type scale by arranging items according to latent variables and using its graphic rating scale to calculate scores. For example, the original NEO Personality Inventory (Costa & McCrae, 1992) uses a Likert-type scale to measure five different types of personality traits and the Work Value Assembly (Sung, Chang, Cheng, & Tien, 2017) uses a Likert-type scale to measure seven dimensions of work values. We can replace the Likert-type scale with a VAS-RRP by forming testlets with five items corresponding to each of the five personality types and seven dimensions of work values.

As well as discovering diverse possible applications for VAS-RRPs, this study suggests several avenues of future research. The first is related to the functions of VAS-RRPs. VAS-RRPs incorporate forced choice into a testlet design to try to reduce or prevent response styles and socially desirable responses (or faking). But several issues remain to be clarified. Are the forced-choice scores of the VAS-RRP more precise than those from VAS (i.e., a single-item VAS-RRP) rating scales? Participants may have more difficulty comparing large numbers of items at once, thus reducing precision. The optimal number of items on a testlet, then, remains an important research question. Additionally, whether ranking or comparisons really reduce or prevent socially desirable responses from over-occurring also remains an open question, and further research should be conducted to test this. Finally, the original VAS format does not include a midpoint. The addition of a midpoint to the VAS-RRP may have distorted participants’ responses. How much, if any, distortion was created is an issue. Another issue is the nonoverlapping requirement for exerting forced-choice function in the VASRRP format. Will rating behaviors be affected by the forced nonoverlapping of specific positions on the line continuum? If we investigate these problems, we could provide more evidence for when and how using the VAS-RRP is most advantageous. Another avenue will be to compare the functionality of the VAS and the VAS-RRP. Despite the finding that the VAS-RRP may elicit less leniency bias and deeper engagement than the VAS, the VAS-RRP did not show higher reliability than the VAS. More different types (e.g., different items in a testlet or different psychological constructs) of VAS-RRP need to be compared with VASs to reveal their actual differences. Future research could also compare differences in bias, validity, and reliability between scaled scores obtained by using IRT models to scale VAS-RRP scores and the original, nonscaled VAS-RRP scores. Finally, it would be worthwhile to investigate methods of strengthening VAS-RRP data analysis. For example, the CTCU model is not the only one that can be employed to process context effects; the correlated-traits–uncorrelated-methods model for processing MTMM data, or the correlated-traits–correlated-methods model (Widaman, 1985) could also be adopted for the analysis of VAS-RRP data. Further comparisons of the pros and cons of these different models will be required.
